# Multimodal management of ectopic hepatic pregnancy: a systematic review of the literature

**DOI:** 10.1007/s00404-024-07739-0

**Published:** 2024-10-01

**Authors:** Maximilian Heinz Beck, Jalid Sehouli, Jonas Alexander Leppig, Sebastian Knitter, Johann Pratschke, Felix Krenzien

**Affiliations:** 1 Department of Gynecology, European Competence Center for Ovarian Cancer, Campus Virchow Klinikum, Charité Universitätsmedizin Berlin, Corporate member of Freie Universität Berlin, Humboldt-Universität Zu Berlin, Berlin Institute of Health, Berlin, Germany; 2 Department of Radiology, Campus Virchow Klinikum, Charité Universitätsmedizin Berlin, corporate member of Freie Universität Berlin and Humboldt-Universität Zu Berlin, Berlin, Germany; 3 Department of Surgery, Campus Virchow Klinikum, Charité Universitätsmedizin Berlin, corporate member of Freie Universität Berlin and Humboldt-Universität Zu Berlin, Berlin, Germany; 4 Berlin Institute of Health at Charité Universitätsmedizin Berlin, BIH Biomedical Innovation Academy, BIH Charité Clinician Scientist Program, Charitéplatz 1, 10117 Berlin, Germany

**Keywords:** Ectopic pregnancy, Abdominal pregnancy, Hepatic pregnancy, Liver pregnancy, Methotrexate

## Abstract

**Purpose:**

Ectopic pregnancies with implantation in the upper abdomen are exceptionally rare. Here we provide a systematic review of hepatic ectopic pregnancies and the corresponding management strategies. Furthermore, this report details a case of ectopic hepatic pregnancy, successfully treated with primary methotrexate (MTX) followed by a two-staged robotic-assisted resection.

**Methods:**

Two independent investigators performed a systematic review using the online search engine PubMed and MEDLINE database. The search utilized the following terms: ‘Hepatic Ectopic Pregnancy,’ ‘Hepatic Extrauterine Pregnancy,’ ‘Hepatic Abdominal Pregnancy,’ and ‘Ectopic Liver Pregnancy.’ Cross-referencing was employed to identify possible additional publications.

**Findings:**

Forty-seven case reports on hepatic pregnancies were identified. Of these, 40 provided manuscripts in the English language. Most patients with hepatic pregnancy presented with mild to moderate abdominal pain, while only a minority exhibited signs of hemodynamically relevant intraperitoneal hemorrhage. Most cases were managed through open surgical removal, although in recent years, there has been an increase in laparoscopically managed cases. Conservative approaches using methotrexate are seldom employed.

**Conclusion:**

Hepatic pregnancies present a rare and challenging clinical scenario. Until now, these cases have usually been treated primarily with open explorative surgery. As reported in this case, primary conservative treatment approaches with MTX before surgery hold promise as a strategy to reduce surgery-related bleeding and morbidity, particularly for asymptomatic or oligosymptomatic patients.

**Supplementary Information:**

The online version contains supplementary material available at 10.1007/s00404-024-07739-0.

## Introduction

Ectopic pregnancies occur in 1–2% of naturally conceived pregnancies [1]. In up to 95% of cases the ectopic pregnancy is located within the fallopian tubes [2]. Non-tubal implantation sites are rare and include ovarian, cervical, interstitial, respectively, cornual, cesarean scar or abdominal pregnancies [3, 4]. Abdominal pregnancies account thereby only for about 1% of all ectopic pregnancies [2, 4]. Intraperitoneal implantation sites can range from the peritoneal surface [5–7] to the involvement of intra-abdominal organs such as the omentum [5, 7–9], intestine [7, 10, 11], spleen [12], diaphragm [11, 13] or liver [14]. These unconventional gestational locations present distinctive challenges, particularly in terms of diagnosis. The rarity and atypical presentation of such cases not only complicate the diagnostic work-up but also pose difficulties in devising appropriate treatment recommendations for the patients. If an intra-abdominal pregnancy is suspected, surgical exploration is usually recommended, given the potential for severe complications, including fatal maternal hemorrhage [3]. While pelvic implantation is more commonly reported, manifestations in the upper abdomen are exceedingly rare, with very limited evidence confined to case reports [15–17]. At present, there exists no standardized diagnostic or therapeutic protocol. In this context, we provide a comprehensive literature review on hepatic ectopic pregnancies alongside a case involving a 24-year-old patient managed through a two-staged approach.

### Clinical scenario

A 24-year-old female patient presented with complaints of pain in the upper right side of her abdomen in the emergency department of our clinic. The cardiopulmonary stable patient had no history of gynecologic morbidities and no known previous pregnancies. She used a non-hormonal intrauterine device (IUD) for contraception. A positive beta-human chorionic gonadotropin (β-hCG) result was detected in the patient's urine upon admission. Clinical examination identified right upper abdominal pain with localized tenderness.

**Fig. 1:**
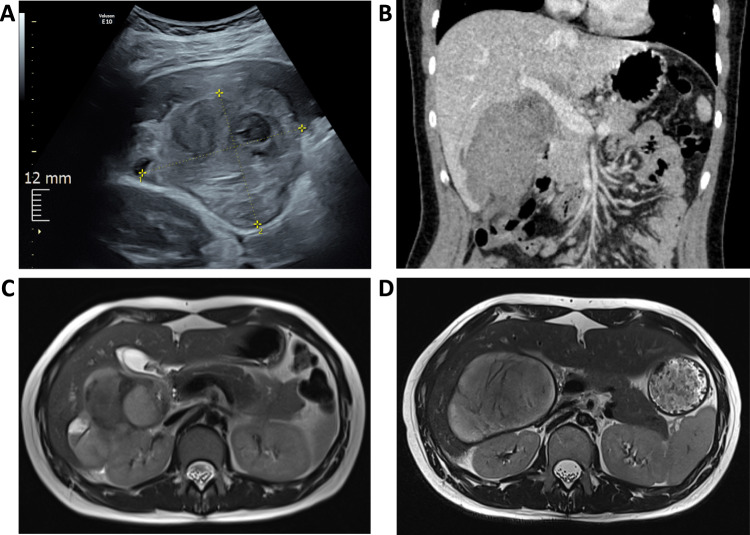
Imaging studies of the 24-year-old patient presenting with hepatic pregnancy. **A** Emergency transabdominal ultrasound on primary presentation showed a 9 × 5 × 4 cm inhomogeneously hyperechogenic tumor with hypoechogenic circumscribed areas on the right hepatic lobe. **B** Contrast-enhanced abdominal CT, coronal plane, exhibiting an intermediate- to low-density tumor measuring approximately 10 × 7 × 11 cm, attached to the right hepatic lobe with hilar involvement. **C** 1.5-Tesla-MRI, T2-HASTE, axial plane, displaying an 11 × 6 cm lobulated inhomogeneous tumor with solid and liquid components located in the Morison pouch. **D** 3-Tesla-MRI, T2-HASTE, axial plane, shows an increasingly circumscribed, roundish tumor with intermediate T2 internal signal, T2 hypointense encapsulation and septa-like central parts. The tumor has remained stable in size

Laboratory analysis showed a β-hCG serum level of 3702 IU/L, moderate leukocytosis (11.39/nL), an elevated CRP (57.8 mg/L), and a hemoglobin level of 11.8 g/dL. Abdominal emergency sonography uncovered an inhomogeneous tumor measuring 9 × 5 × 4 cm, attached to the right lobe of the liver, with no significant internal perfusion (Fig. 1). Transvaginal sonography indicated a properly positioned IUD in a line-shaped uterine cavity, with normal adnexa showing age-appropriate follicular coverage and no evidence for tubal ectopic pregnancy. A moderate amount of echogenic free fluid was present in the rectouterine and vesicouterine pouch, fluid in the upper abdomen was absent. An additional computer tomography (CT) scan disclosed a 10 × 7 cm (sub-) hepatic tumor, consistent with a suspected subcapsular hepatic hematoma (Fig. 1).

The patient was admitted to the gynecologic department for further assessment. Primary considerations included abdominal pregnancy with (sub-) hepatic implantation, hemorrhaged β-HCG-producing hepatic tumor, and secondarily, chorionic carcinoma. For further work-up and differential diagnosis, tumor markers (AFP, CA 125, HE4, CA 15–3, CA 19–9, CA 72–4, and CEA) were assessed, all of which were found to be within normal limits for the patient's age. Additional magnetic-resonance imaging (MRI) of the abdomen was conducted, revealing a lobulated, inhomogeneous mass (113 × 58 mm) in Morison’s pouch with partial hyperintensity in native T1 weighting and pelotting of segment V and VI, see also Fig. 1.

**Fig. 2:**
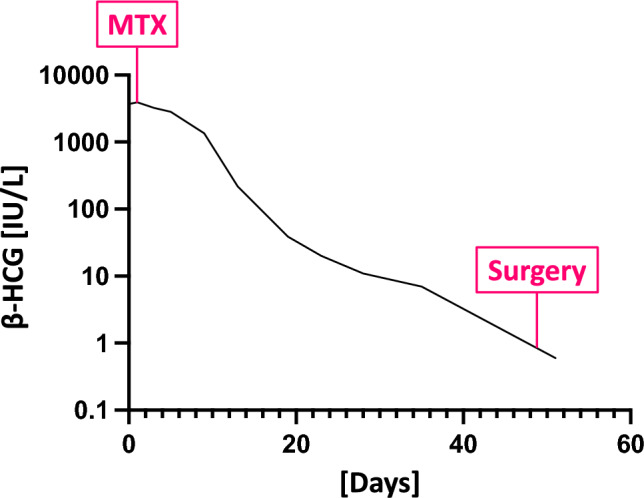
Time course of serum β-hCG levels. The y-axis displays the β-hCG serum levels on a logarithmic scale, while the x-axis represents the timeline in days. The time points of methotrexate (MTX) administration and surgery are indicated by magenta-colored boxes

Based on the findings, a clinical diagnosis of abdominal hepatic ectopic pregnancy was made, and a primarily conservative approach was discussed and agreed upon with the patient. A single dose of methotrexate (50 mg/m^2^ body surface) in off-label use was administered, resulting in a decline in β-hCG levels (see Fig. 2). The patient was discharged after 6 days of uneventful observation.

β-hCG levels continued to decrease subsequently over weeks. Follow-up ultrasound examinations were conducted, and no significant alterations in lesion size were observed. In line with the sonographic results, a follow-up MRI after one month unveiled a tumor undergoing liquefaction while maintaining a relatively stable size (see Fig. 1). Due to persistent symptoms and the absence of significant tumor shrinkage, the possibility of secondary surgical tumor resection was discussed with the hepatobiliary surgery department. Eight weeks following MTX administration, a minimal-invasive robotic-assisted tumor resection was performed by the hepatobiliary surgery team. Intraoperatively, a cystic tumor adjacent to the gallbladder and the ligamentum hepatoduodenale with substantial adhesions to the greater omentum and infiltration of the right liver lobe was identified (see Video 1). Local tumor resection, including an atypical resection of liver segments V and VI, was carried out, necessitating cholecystectomy for complete removal. Importantly, the anatomical structures, including the portal vein, hepatic artery, and bile duct remained intact. The cystic tumor was safely removed without any severe bleeding. The patient's postoperative course was uneventful, leading to discharge after five days. Final histopathological examination unveiled chorionic villi in a state of severe regression confirming the diagnosis of abdominal ectopic gravidity.

## Material and methods

A systematic review was conducted with the use of the online search engine *PubMed* and MEDLINE database. The following search terms were used to identify cases of hepatic ectopic pregnancies: Hepatic Ectopic Pregnancy’; Hepatic Extrauterine Pregnancy’; Hepatic Abdominal Pregnancy’; Ectopic Liver Pregnancy’. All case reports were included in the analysis until December 2023. Literature search and review of the abstracts was carried out by two independent investigators. Case reports were only considered if available in the English language. References of identified manuscripts were checked for potentially missed additional reports. The patient gave her written consent for publication of their case.

## Results

Based on the search terms mentioned above, we were able to identify 47 case reports of abdominal ectopic pregnancies with hepatic implantation sites, published between 1956 and 2023. Of these, 40 provided manuscripts in the English language [14, 16, 18–55]. Two of these reports, identified by cross-referencing, were not listed in *PubMed* [53, 54]. Seven non-English publications were not considered for further review [56–62].

Table 1 provides an overview of the identified reports. The median patient age at presentation was 31 years (range 18–46) and the median gestational age was 9 weeks (range 4–36) at the time of diagnosis, with 10 cases missing gestational week information and one case missing age information. In six cases (15.4%), the pregnancy continued beyond the first trimester [11, 19, 20, 34, 39, 41] and in three cases (7.5%) the hepatic pregnancy resulted in a live birth [19, 39, 41].

**Table 1:** Overview of 40 case reports on hepatic pregnancies, published between 1956 and 2023

References	Age	GA	Localization	Clinical presentation				Clinical Management				Outcome	Special Characteristics
				Pain	Hem	Shock	Other	LPM	LSC	MTX	Other		
Murley [36]	39	N/A	Right lobe	Yes	Yes	Yes	No	Yes	No	No	No	Cure	No
Kirby [18]	26	8–10	Right lobe	Yes	Yes	No	Dyspnea	Yes	No	No	No	Cure	No
Luwuliza Kirunda [23]	40	N/A	Right lobe	No	No	No	Abdominal mass	Yes	No	No	No	Cure	Lithopedion
Krause [32]	26	N/A	Right lobe	Yes	Yes	Yes	No	Yes	No	No	No	Cure	No
Hietala 31	23	N/A	N/A	Yes	Yes	N/A	N/A	Yes	No	No	No	Cure	N/A
Mitchell 14	20	N/A	Right lobe	Yes	Yes	Yes	Vomiting	Yes	No	No	No	Cure	No
Shukla 19	25	28	Right lobe	Yes	No	No	No	Yes	No	Postop	No	Cure, live birth (1300 g)	Neonatal demise. Placenta remained
Veress 22	31	4	Right lobe	Yes	Yes	No	No	Yes	No	No	No	Cure	No
Børlum 45	23	9	Right lobe	Yes	Yes	Yes	No	Yes	No	No	No	Cure	No
Harris 34	23	14	Right lobe	No	No	No	No	Yes	No	No	No	Cure	No
de Freitas 25	32	8	Right lobe	Yes	Yes	Yes	No	Yes	No	No	No	Cure	No
Nichols 26	32	N/A	Porta hepatis	Yes	Yes	No	Irregular bleeding, vomiting	Yes	No*	Intraop	No	Cure	No
Delabrousse 46	46	11–12	Right lobe	Yes	Yes	Yes	No	Yes*	No	Postop	No	Cure	No
Chui 21	35	8	Caudate lobe	Yes	Yes	No	No	Yes	No	No	No	Cure	Abdominal packing
Shippey, 29	N/A	11	Right lobe	Yes	No	No	No	No*	No*	Primary	Fetocide w/KCI	Cure	No
Chin 35	30	4	Porta hepatis	Yes	Yes	No	No	No	Yes	Postop	No	Cure	No
Moores 40	23	12	Gallbladder	Yes	No	No	Nausea	No	No	Primary	Fetocide w/KCI	Cure	No
Ramphal 39	18	19	Porta hepatis	No	No	No	Abdominal mass	Yes	No	No	No	Cure, live birth (1800 g)	Placenta was not removed
Wang 44	33	7	Left lobe	Yes	Yes	No	Irregular bleeding	Yes	No	No	No	Cure	No
Yadav 20	25	18	Right lobe	Yes	Yes	Yes	Vomiting	Yes	No	No	Hep. Artery Embol	Maternal Death	DIC; Multiorgan failure
Kuai 30	33	5	Right lobe Subdiaphragm	Yes	Yes	No	No	Yes	No	No	No	Cure	No
Ma 37	31	8	Right lobe	Yes	Yes	Yes	No	Yes	No	Postop	TACE	Cure	No
Qiao 42	31	10	Right lobe	No	No	No	No	Yes	No	No	Poly-CTx	Cure	No
Hu 28	32	8	Right lobe	Yes	No	No	No	Yes	No	No	No	Cure	No
Brouard 41	20	36	Right lobe	Yes	No	No	No	Yes	No	No	No	Cure, live birth (2800 g)	Placenta remained
Hao 33	31	N/A	Right lobe	Yes	No	No	No	Yes	No	No	No	Cure	N/A
Guo 55	31	N/A	Right lobe	No	No	No	Abdominal distention	Yes	No	No	No	Cure	No
Tu 24	33	N/A	Right lobe	Yes	No	No	No	Yes	No	No	No	Cure	N/A
Cai 43	31	6	Right lobe	No	No	No	Abdominal distention	No	Yes	No	No	Cure	No
Sibetcheu 38	24	9	Right lobe	Yes	No	No	No	No	No	Primary	No	Cure	No
Wang 27	31	6	Right lobe	No	No	No	No	Yes	No	No	No	Cure	No
Zhao 47	21	14	Left lobe	No	No	No	Irregular bleeding	No	Yes	Primary	No	Cure	No
Garzon 16	37	9	Right lobe	Yes	Yes	No	No	No	Yes*	No	No	Cure	No
Yin 49	28	5	Right lobe	Yes	Yes	No	No	N/A	N/A	No	No	Cure	No
He 51	23	N/A	Right lobe	Yes	No	No	N/A	Yes	No	No	No	Cure	No
Srdan 53	40	6	Right lobe	Yes	Yes	Yes	No	Yes	No*	No	No	Cure	No
Zhang 50	30	9	Right lobe	Yes	Yes	No	No	Yes	No	No	No	Cure	No
Li 54	30	9	Right lobe	No	No	No	Irregular bleeding	Yes	No	No	No	Cure	No
Rajanbabu 48	33	N/A	Right lobe	Yes	No	No	No	No	Yes*	No	No	Cure	No
Xu 52	29	7	Right lobe	Yes	No	No	No	No	Yes	No	No	Cure	No

*CTx* Chemotherapy, *GA* Gestational Age at diagnosis, *Embol*. Embolization, *Hem* Hemoperitoneum, *hep*. Hepatic, *inj*. Injection, *Intraop*. Intraoperative, *KCl* Potassium Chloride, *LPM* Laparotomy, *LSC* Laparoscopy, *N/A* not available, *Postop*. Postoperative, *Subdiaphragm*. Subdiaphragmatic, *TACE* Trans Arterial Chemo-Embolization, *w*/ with, *US* ultrasound

^*^Initially performed and not leading to diagnosis. Secondary intervention was performed

The majority of hepatic implantations were described on the inferior side of the right hepatic lobe (*n* = 31; 77.5%). Implantation on the left hepatic lobe (*n* = 2; 5%), the caudate lobe (*n* = 1; 2.5%), or between the right hepatic lobe and diaphragm (*n* = 1; 2.5%) were observed only in a limited number of cases. [21, 30, 44, 47]. Implantation on the liver hilum was described in three cases [26, 35, 39] and one report observed additional attachment to the gallbladder [40]. Table 1 presents an overview of the implantation sites.

Most patients presented with (sub-)acute abdominal pain and right upper abdominal tenderness. Some patients reported additional symptoms such as amenorrhea, irregular vaginal bleeding, abdominal distention, epigastric discomfort, nausea or vomiting [14, 20, 39, 40, 47, 54]. A subset of cases presented with either minimal or absent symptoms and were primarily diagnosed by positive β-hCG testing. Notably, patients did not report any pain in 9 cases (22.5%). Conversely, hemodynamic instability and clinical manifestations of hypovolemic shock were evident in 9 (22.5%) cases. Table 1 provides a comprehensive delineation of clinical symptoms. In three cases (7.5%), the hepatic pregnancy was reported with an IUD in situ [43, 45, 49].

Transvaginal ultrasound (TVUS) was mostly employed as the initial diagnostic tool for suspected ectopic pregnancy. Common findings included unremarkable adnexa, a potentially enlarged uterus with variable endometrial thickness, and the presence of free fluid in the cul-de-sac. Abdominal ultrasound studies typically revealed hypodense cystic lesions surrounded by a hyperechoic ring or lesions of mixed echogenicity. Doppler sonography depicted peripheral hypervascularization [16, 30, 33, 43, 50, 54, 55]. Due to the challenging nature of the diagnosis, additional imaging modalities such as CT and/or MRI were performed in most cases. In 45% (*n* = 18) of cases, the diagnosis of hepatic pregnancy was made by clinical examination and imaging before any surgical intervention [19–21, 23, 24, 27, 33, 34, 38–41, 43, 47, 50, 52, 54, 55]. Fetal structures were identified in imaging in 27.5% (*n* = 11) of reported cases [19, 20, 28, 29, 34, 39–41, 46, 47, 52]. Notably, two authors have reported the use of 18F-FDG PET-CT as a supplementary diagnostic study [33, 43].

Surgical removal of hepatic pregnancies was performed in most cases (92.5%; *n* = 37). An open surgical approach was more commonly selected (75%; *n* = 30). In recent years, there has been an increase in laparoscopic approaches. In six cases, either laparoscopy [16, 26, 48] or pelvic laparotomy [29, 46, 53] was initially conducted without immediate identification of the hepatic pregnancy. In these cases, secondary interventions or further diagnostic were necessitated by increasing β-hCG levels, persistent or worsening pain, or severe hemorrhage, ultimately leading to the diagnosis of hepatic pregnancy. Five of these cases underwent subsequent surgical interventions, whereas one case was managed by following MTX treatment [29].

MTX was administered in 9 cases (22.5%) for the treatment of hepatic pregnancy, either as a primary conservative approach without surgical removal [29, 38, 40] or as a multimodal approach in combination with surgical resection [19, 26, 35, 37, 46, 47]. In multimodal approaches, MTX administration varied. It was either administered immediately prior to surgery [47], intraoperatively (20 mg MTX) in case of implantation on the porta hepatis and limited resectability [26] or postoperatively in cases with incomplete removal due to limited operability or increasing postoperative β-hCG levels [19, 35, 37, 46]. In a case marked by postoperative elevation of β-hCG levels, a successful postoperative transarterial chemoembolization (TACE) was reported. This involved the utilization of a lipiodol-methotrexate (1 mg/kg) emulsion, followed by gelatine sponge particle-based embolization [37].

Three primary conservative approaches were reported [29, 38, 40]. In one instance, a combination of MTX (1 mg/kg intramuscularly) and 300 mg mifepristone was given [38]. The other two cases of conservative management utilized a combination of systemic MTX administration and ultrasound-guided intrathoracic fetocide using potassium chloride [29, 40]. Follow-up sonography was conducted in two of these cases, revealing no remnants of the hepatic pregnancy after one year [29] and 17 months, respectively [40].

A broad variety of MTX dosage regimens was reported, including both single [19, 35, 38] and multidose administrations [29, 40, 46]. In one case, the misdiagnosis of a suspected chorioncarcinoma resulted in the administration of polychemotherapy (VCR/VP16/KSM/5-FU). During a follow-up sonography, a vital fetus was visualized and surgical removal of the hepatic pregnancy was subsequently performed [42].

The majority of cases were managed successfully by the aforementioned therapeutic approaches. Reports on estimated blood loss were available only in a minority of case reports, varying from 100 to 4000 mL. Notably, in ten cases (25%), the total blood loss surpassed 1000 mL [14, 16, 20, 21, 28, 32, 37, 44, 45, 47]. One patient succumbed due to hypovolemic shock leading to multiorgan failure. Despite repeated laparotomies, abdominal packing, and hepatic artery embolization, bleeding in this case could not be effectively controlled [20].

In three instances, hepatic pregnancies resulted in live births. In one case, the pregnancy was diagnosed at 28 weeks of gestation, leading to a laparotomy with the delivery of a vital male neonate. Neonatal demise occurred after 30 min under unknown circumstances [19]. In two cases, healthy neonates were successfully delivered [39, 41]. In the first case, an unmonitored pregnancy was diagnosed at 36 weeks of gestation, resulting in the delivery of a 2970 g healthy female fetus through a midline laparotomy [41]. In the second case, the diagnosis was made at 19 weeks of gestation, and an observational approach was adopted until sufficient fetal maturity was attained. At 34 weeks of gestation, a healthy 1800 g neonate was delivered through a midline laparotomy [39]. Remarkably, in all three cases, the placenta was not removed. Follow-up MRI revealed diminishing residuals after 7 weeks [41] and, respectively, 14 months [39]. In the case of a 40-year-old oligosymptomatic patient, a hepatic lithopedian measuring 35 cm in length was surgically removed. The abdominal mass was noted subsequent to a live birth. The authors suspected a previous simultaneous uterine and hepatic pregnancy in this case [23].

## Discussion

Hepatic pregnancies are rare and present diagnostic and therapeutic challenges due to their infrequent and atypical manifestation as an ectopic pregnancy. In this comprehensive literature review, we identified 47 published cases of abdominal pregnancies with hepatic implantation. In the majority of reported cases, primary surgical removal via laparotomy was pursued, while conservative or multimodal approaches utilizing MTX were reported only in a few cases. Here, we present the first case of successful multimodal management for a hepatic pregnancy, involving initial MTX treatment followed by robotic-assisted resection.

In our perspective, primary MTX treatment for hepatic or abdominal pregnancies is a valuable therapeutic option for selected patients exhibiting no or only moderate symptoms, provided that the diagnosis can be made by clinical examination and imaging. An interdisciplinary approach, closely coordinated with radiologists and general or visceral surgeons, should be adopted in such cases.

As evidenced by this literature review, only a minority of patients present with hemodynamically relevant bleeding, suggesting that a primarily conservative or multimodal approach might be suitable for a significant number of patients with hepatic (or abdominal) pregnancies. Subsequent minimal-invasive removal of remaining avital pregnancy tissue is likely associated with less surgical trauma and fewer bleeding complications. Notably, the literature reports three cases of successful conservative approaches without any surgical removal [29, 38, 40], prompting the question of whether secondary removal of remaining pregnancy material is necessitated at all in asymptomatic or minimally symptomatic patients. In our case, the decision for secondary removal of the hepatic pregnancy was based on moderate but persistent symptoms and a hepatic lesion that was MRI-morphologically constant in size, alongside concerns about potential secondary complications such as superinfection or major adhesions. Nevertheless, complete sonomorphologic resorption of the hepatic pregnancy is possible and was reported in primarily conservatively managed cases after one year [29, 40], and similarly, extensive resorption of placental tissue occurred after reported live births in which the hepatic placental bed was left in place [39, 41]. Based on these reports, secondary surgery or observational strategies should be discussed with the patient after primary MTX therapy. The resorption of the pregnancy can be monitored by consecutive β-hCG determinations, as exemplified in this case, along with follow-up imaging [63]. As far as our knowledge extends, only one case involving primary MTX administration followed by subsequent surgery has been reported to date [47]. MTX was given preoperatively in this case, and during the subsequent removal of the placenta massive hemorrhage occurred with a reported blood loss of 2000 mL. Due to the short interval between MTX administration and surgery, there was presumably no relevant degeneration of the hepatic pregnancy, and longer intervals until surgical removal should be considered.

Cytostatic treatment as a primary approach has consistently been reported as effective for managing other rare ectopic pregnancies such as cesarean scar, cervical, cornual, or ovarian pregnancies [64, 65]. For hemodynamically stable patients with cesarean scar pregnancies, a single-dose MTX regimen (50 mg) is recommended when the gestational age is under 8 weeks, serum hCG is below 5000 IU/L, the gestational sac measures less than 2.5 cm, and/or there is no evidence of embryonic cardiac activity [64]. In cervical pregnancies, treatment failure has been linked to factors such as serum hCG levels exceeding 10,000 IU/L, the presence of cardiac activity, and a crown-rump length greater than 10 mm [66]. While these criteria may not be directly applicable to abdominal or hepatic pregnancies, they offer some guidance in identifying patients who may be candidates for drug-based treatment.

Our proposed conservative approach may only be feasible when imaging studies provide sufficient certainty for the complex and challenging diagnosis of hepatic pregnancy. As demonstrated in this systematic review, the diagnosis of hepatic pregnancy was made through clinical examination and imaging in roughly half of the cases.

The pathogenesis of hepatic pregnancies remains speculative. In the literature, a distinction is drawn between primary and secondary abdominal pregnancies. In primary abdominal pregnancies, direct intraperitoneal implantation is presumed, while secondary abdominal pregnancies, presumably more frequent, are believed to be preceded by tubal abortion or early tubal or cornual rupture with subsequent reimplantation in the abdominal cavity [3, 4]. Due to the anatomic proximity, pelvic implantation is the most common implantation site for abdominal pregnancies [5]. Nevertheless, the liver, with its robust perfusion and large organ size, appears to be a favorable site for implantation. The clockwise peristalsis could facilitate the passive transportation of the fertilized ovum to the upper abdomen and subsequent implantation on the exposed lower surface of the right hepatic lobe.

Risk factors for abdominal pregnancy are believed to correspond to the generally known risk factors for ectopic pregnancies and include previous tubal surgery, previous ectopic pregnancies, assisted conception, current use of a contraceptive uterine device or history of smoking [1, 3].

## Conclusion

Hepatic pregnancies present a rare and challenging clinical scenario. Until now, these cases have usually been treated primarily with surgery. In our case report we present the first case, to our knowledge, of a two-staged approach of MTX administration followed by robotic-assisted resection. Considering the substantial risk of perioperative bleeding, treatment with MTX before surgery holds promise as a strategy to prevent bleeding situations, particularly for asymptomatic or oligosymptomatic patients. This approach is viable provided that the diagnosis can be accurately determined through clinical examination and imaging.

## Supplementary Information

Below is the link to the electronic supplementary material.

Supplementary file1 (PNG 6445 KB)

Supplementary file2 (MP4 99039 KB)
