# Supplementary figures and images for: Multimodal management of ectopic hepatic pregnancy: a systematic review of the literature
Source: Arch Gynecol Obstet. 2024 Oct 1;310(5):2345–53. doi: 10.1007/s00404-024-07739-0 (PMC11485115; doi:10.1007/s00404-024-07739-0)

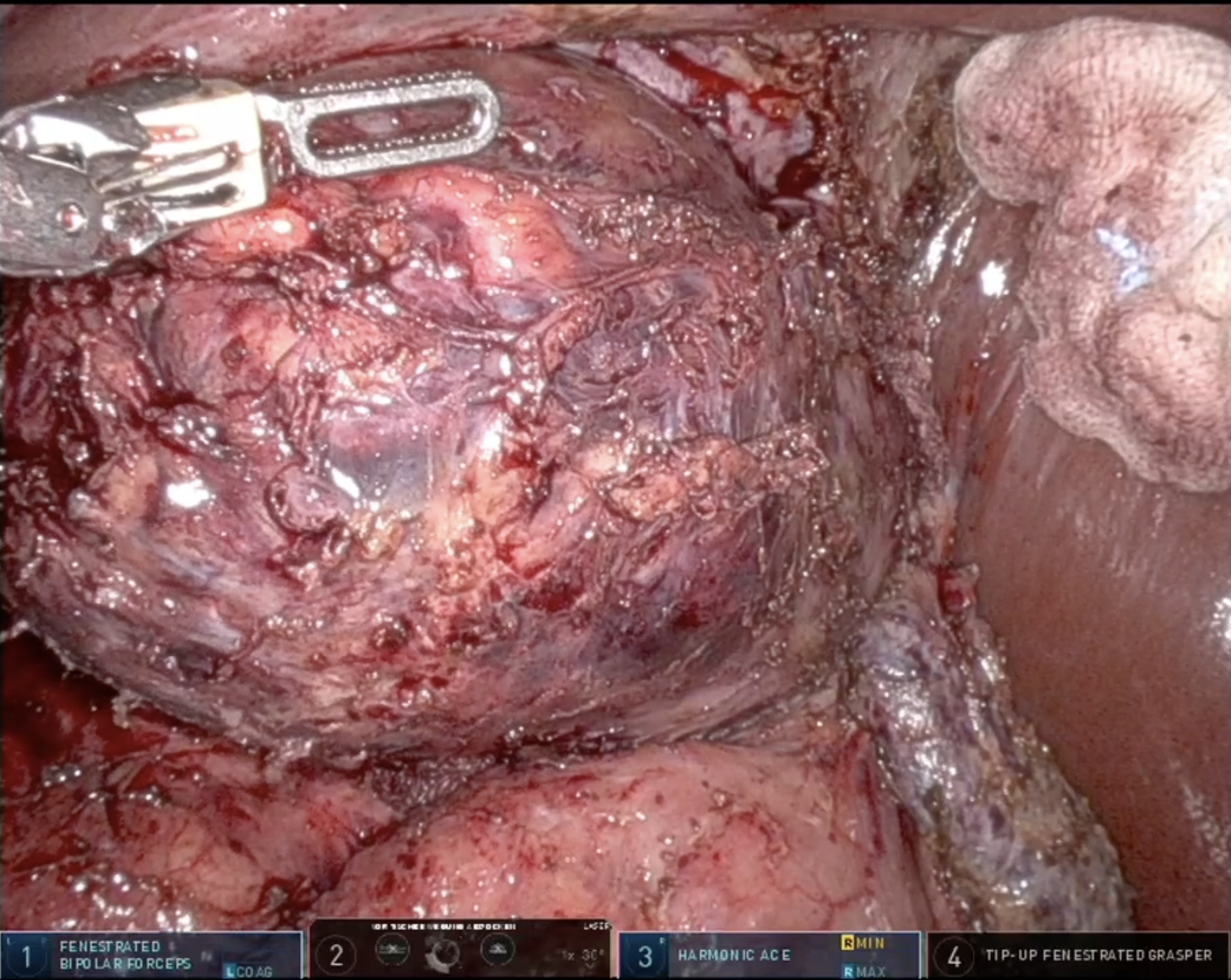

Supplement: Supplementary file 1 — Supplementary file1 (PNG 6445 KB) [file 404_2024_7739_MOESM1_ESM.png]
